# Diagnostic accuracy of coronary computed tomography angiography-derived fractional flow reserve (CT-FFR) in patients before liver transplantation using CT-FFR machine learning algorithm

**DOI:** 10.1007/s00330-022-08921-1

**Published:** 2022-06-22

**Authors:** Maximilian Schuessler, Fuat Saner, Fadi Al-Rashid, Thomas Schlosser

**Affiliations:** 1 Institute of Diagnostic and Interventional Radiology and Neuroradiology, University Hospital Essen, Essen, Germany; 2 Department of General-, Visceral- and Transplantation Surgery, University Hospital Essen, Essen, Germany; 3 Department of Cardiology and Vascular Medicine, West German Heart and Vascular Center, University Hospital Essen, Essen, Germany

**Keywords:** Machine learning, Fractional flow reserve, Computed tomography, Coronary angiography, Liver transplantation

## Abstract

**Objectives:**

Liver transplantation (LT) is associated with high stress on the cardiovascular system. Ruling out coronary artery disease (CAD) is an important part of evaluation for LT. The aim of our study was to assess whether CT-derived fractional flow reserve (CT-FFR) allows for differentiation of hemodynamically significant and non-significant coronary stenosis in patients evaluated for LT.

**Methods:**

In total, 201 patients undergoing LT evaluation were included in the study. The patients received coronary computed tomography angiography (CCTA) to rule out CAD and invasive coronary angiography (ICA) to further evaluate coronary lesions found in CCTA if a significant (≥ 50 % on CCTA) stenosis was suspected. CT-FFR was computed from CCTA datasets using a machine learning–based algorithm and compared to ICA as a standard of reference. Coronary lesions with CT-FFR ≤ 0.80 were defined as hemodynamically significant.

**Results:**

In 127 of 201 patients (63%), an obstructive CAD was ruled out by CCTA. In the remaining 74 patients (37%), at least one significant stenosis was suspected in CCTA. Compared to ICA, sensitivity, specificity, PPV, and NPV of the CT-FFR measurements were 71% (49–92%), 90% (82–98%), 67% (45–88%), and 91% (84–99%), respectively. The diagnostic accuracy was 85% (85–86%). In 69% of cases (52 of 75 lesions), additional analysis by CT-FFR correctly excluded the hemodynamic significance of the stenosis.

**Conclusions:**

Machine learning–based CT-FFR seems to be a very promising noninvasive approach for exclusion of hemodynamic significant coronary stenoses in patients undergoing evaluation for LT and could help to reduce the rate of invasive coronary angiography in this high-risk population.

**Key Points:**

*• Machine learning–based computed tomography-derived fractional flow reserve (CT-FFR) seems to be a very promising noninvasive approach for exclusion of hemodynamic significance of coronary stenoses in patients undergoing evaluation for liver transplantation and could help to reduce the rate of invasive coronary angiography in this high-risk population.*

## Introduction

Liver transplantation (LT) is associated with high stress on the cardiovascular system; cardiovascular complications can occur intraoperatively, early in the posttransplant period or later in follow-up [1, 2]. Since the prevalence of coronary artery disease (CAD) is relatively high in liver transplantation candidates [3], invasive coronary angiography (ICA) has become the standard examination to rule out coronary artery disease during the preoperative screening process in patients with a higher risk for many transplantation centers. While ICA represents the standard of reference for ruling out CAD and the quantification of coronary artery stenoses, for example by assessment of fractional flow reserve (FFR) [4], the major drawback of this method is its invasiveness with associated risks [5]. Coronary computed tomography angiography (CCTA) is an alternative non-invasive method for ruling out coronary disease [6, 7] in patients with a low clinical likelihood of CAD or patients with contraindication for ICA. However, CCTA primarily provides anatomic and morphologic information about coronary arteries and is not able to reliably predict the hemodynamic significance of a coronary lesion [8]. Furthermore, CCTA is known to overestimate the functional severity of a coronary artery lesion [8]. A relatively new method for noninvasive quantification of coronary artery stenosis is the so-called computed tomography–derived FFR (CT-FFR) [9–12]. Here, the FFR is calculated from CCTA data sets using computational fluid dynamics (CFD) or a machine-learning algorithm.

The aim of our study was to evaluate whether the assessment of CT-FFR allows for differentiation of hemodynamically significant and non-significant coronary stenoses in patients undergoing evaluation for LT.

## Material and methods

### Study population and CCTA imaging

This retrospective study was approved by the institutional review board and the requirement to obtain informed consent was waived (21-10191-BO).

Between September 2017 and March 2020, a total of 201 patients (128 men (64%), median age 57 years, mean age 57.4 ± 8.9 years, range 18–87 years, see Table 1) underwent CCTA in our institution for preoperative screening for LT.

**Table 1:** Patient demographics (*N* = 201)

Patient demographics, *N* = 201	
Mean age (SD)	57.4 (8.9)
Gender	*n* (percentage)
Male	128 (64%)
Female	73 (36%)
Liver disease leading to transplantation evaluation	*n* (percentage)
Hepatocellular carcinoma	58 (29%)
Alcoholic cirrhosis	54 (27%)
Biliary cirrhosis (PSC-, PBC-, SSC-, or IgG4-associated cholangitis)	32 (16%)
Viral hepatitis-induced cirrhosis	21 (11%)
Cryptogenic cirrhosis	12 (6%)
Non-alcoholic steatohepatitis cirrhosis	6 (3%)
Cirrhosis caused by autoimmune hepatitis	5 (3%)
Other diseases (A1AD, non-alcoholic drug-induced cirrhosis, M. Wilson, M. Osler, polycystic liver disease, transplant failure)	13 (7%)

*PSC* primary sclerosing cholangitis; *PBC* primary biliary cholangitis; SSC secondary sclerosing cholangitis; *A1AD* alpha 1 antitrypsin deficiency

CCTA was performed using a third-generation dual-source 384-slice CT scanner (SOMATOM Force, Siemens Healthineers). Patients whose heart rates exceeded 65 bpm received up to 20 mg metoprolol intravenously. In addition, two jets of nitroglycerin spray (0.8 mg nitroglycerin) were applied in all patients. Prior to CCTA, a non-contrast scan was performed to determine the coronary calcium score. For CCTA a 70 ml bolus of iodine-containing contrast medium (Ultravist 300, Bayer Vital GmbH) was injected at an injection rate of 6 ml/s, followed by 20 ml of saline chaser at the same injection rate. Data acquisition was performed using a retrospective ECG-gated spiral acquisition with tube current modulation, targeting the 70–80% phase of the R-R interval and the mid-diastolic phase. For patients with a heart rate above 65 bpm, the 50–80% phase of the R-R interval was targeted. The examinations were performed with a reference of 300 mAs and 90 kV. For reporting, multiphase reconstructions in 5% steps of the R-R interval in 0.75-mm slice thickness were made as standard and archived in the PACS (GE Healthcare GmbH). CCTA examinations were evaluated using standardized procedures by two radiologists, a resident, and a board-certified cardiovascular radiologist with more than 15 years of experience. After assessment of the calcium score (Agatston score), visual morphological analysis of the individual coronary vessels was performed with the aid of axial data sets and multiplanar reconstructions as well as 3D imaging with regard to possible coronary anomalies and the presence of coronary plaques and stenoses. Post-processing was done using the software syngo.via (Siemens Healthineers). Coronary stenoses were graded according to the SCCT guidelines for the interpretation and reporting of coronary CT angiography [13] and a stenosis of ≥ 50 % was considered significant.

### Machine learning–based CT-FFR computation

Computation of machine learning–based CT-FFR was performed using a workstation-based, on-site solution (CT-FFR prototype version 3.2 on syngo.via Frontier, Siemens Healthineers) by a radiologist with 3 years of experience in cardiovascular radiology.

The machine learning–based algorithm trained by Siemens Healthineers was described in detail by Itu et al [14]: 12,000 synthetic coronary geometries were generated, for each of which the CT-FFR for the entire coronary artery tree was calculated using a reduced-order computational blood flow model. Subsequently, the machine learning–based algorithm was trained with 10,000 of these datasets using a deep neural network with four hidden layers, and the result was validated against the remaining 2,000 datasets.

The radiologist performing the analysis of CT-FFR was blinded to the results of the ICA. After loading the CCTA data, the centerline of each coronary artery was generated semi-automatically. If necessary, manual adjustment was performed to correct the centerline. In the next step, the software semi-automatically contoured the lumen of the coronary arteries. If necessary, manual adjustment was performed to correct the lumen contour. After confirming the centerline and the lumen contour, the stenotic region of the coronary vessel was defined manually. The final step in the workflow was the automatic computation of the CT-FFR values based on the segmented coronary tree model. The CT-FFR values were visualized as a volume rendering technique image of the ascending aorta and the coronary arteries with a colored representation of the CT-FFR values. By hovering with the mouse over the coronary tree, the CT-FFR values for a specific position could be displayed and saved for further analysis. In this study, the CT-FFR value was measured 1–2 cm distal to the stenosis.

### Invasive catheter angiography with FFR/iFR-measurement

ICA was performed following local standards by an experienced, board-certified cardiologist via a radial or femoral approach including selective catheterization of the coronary arteries. The time interval between CCTA and ICA was between 1 and 4 weeks. The degree of stenosis of the coronary arteries was determined by visual assessment using quantitative coronary angiography (QCA). In addition to visual analysis, fractional flow reserve (iFFR) or instantaneous wave-free ratio (iFR) was determined for coronary lesions with visually uncertain hemodynamic significance, which was defined as coronary stenosis > 50% and < 80% in QCA. In the case of iFFR, adenosine was used as a hyperaemic agent. iFFR values ≤ 0.80 and iFR values ≤ 0.90 were considered hemodynamically significant.

### Statistical analysis

Statistical analysis including the determination of sensitivity, specificity, positive predictive value (PPV), negative predictive value (NPV), and diagnostic accuracy was done using the software R, version 4.1.0 (R Foundation for Statistical Computing) and the software Microsoft Office 365 Excel (Microsoft Corporation).

## Results

The mean Agatston score of all patients was 367 ± 882 (median score 22, range 0–7752). In 127 of 201 patients (63%), an obstructive CAD was ruled out by CCTA. In the remaining 74 patients (37%), at least one significant stenosis was suspected in CCTA; in these patients, CT-FFR was additionally measured and ICA was performed to further assess coronary artery lesions found in CCTA.

**Fig. 1:**
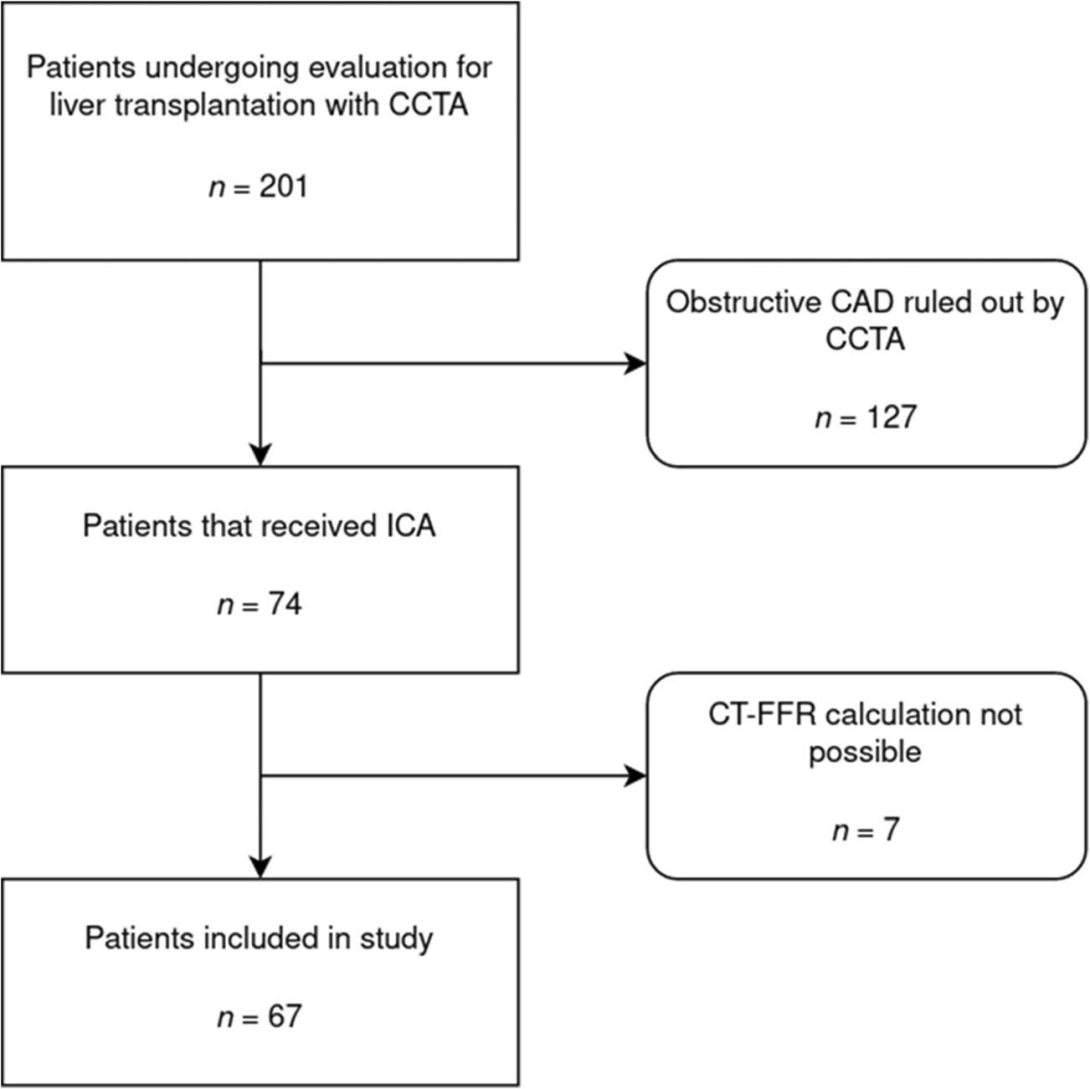
Flow diagram for patient recruitment. *CCTA* coronary computed tomography angiography; *CAD* coronary artery disease; *ICA* invasive coronary angiography; *CT-FFR* computer tomography derived fractional flow reserve

In seven of the 74 patients for which CT-FFR was to be computed (10%), evaluation by the machine-learning-based algorithm was not possible because of the following reasons: (1) unrecognized coronary ostia, most likely due to motion artifacts; (2) insufficient contrast of a coronary artery in the periphery, which was wrongly assessed as total occlusion by the algorithm; (3) venous overlay with incorrect recognition of coronary arteries; (4) severe coronary calcifications with inadequate detection of the midlines; (5) insufficient contrast of the coronary arteries with erroneous detection of the same; (6) high-grade ostial stenosis of the RCA with erroneous detection of the following vessel; (7) noninterpretability in the presence of pulsation artifacts. The respective patients were therefore excluded from the study. Finally, a total of 67 patients (Fig. 1) with a total of 75 coronary lesions (Table 2) were included in the study analysis.

**Table 2:** Lesion characteristics

Lesion characteristics		*N* (%)
Vessel	LAD	36 (48%)
	RCX	15 (20%)
	RCA	24 (32%)
Lesions in LAD	Significant stenosis on ICA	11 (31%)
	No significant stenosis on ICA	25 (69%)
Lesions in RCX	Significant stenosis on ICA	2 (13%)
	No significant stenosis on ICA	13 (87%)
Lesions in RCA	Significant stenosis on ICA	5 (21%)
	No significant stenosis on ICA	19 (79%)

*LAD* left anterior descending artery; *RCA* right coronary artery; *RCX* ramus circumflex artery; *ICA* invasive coronary angiography

**Fig. 2:**
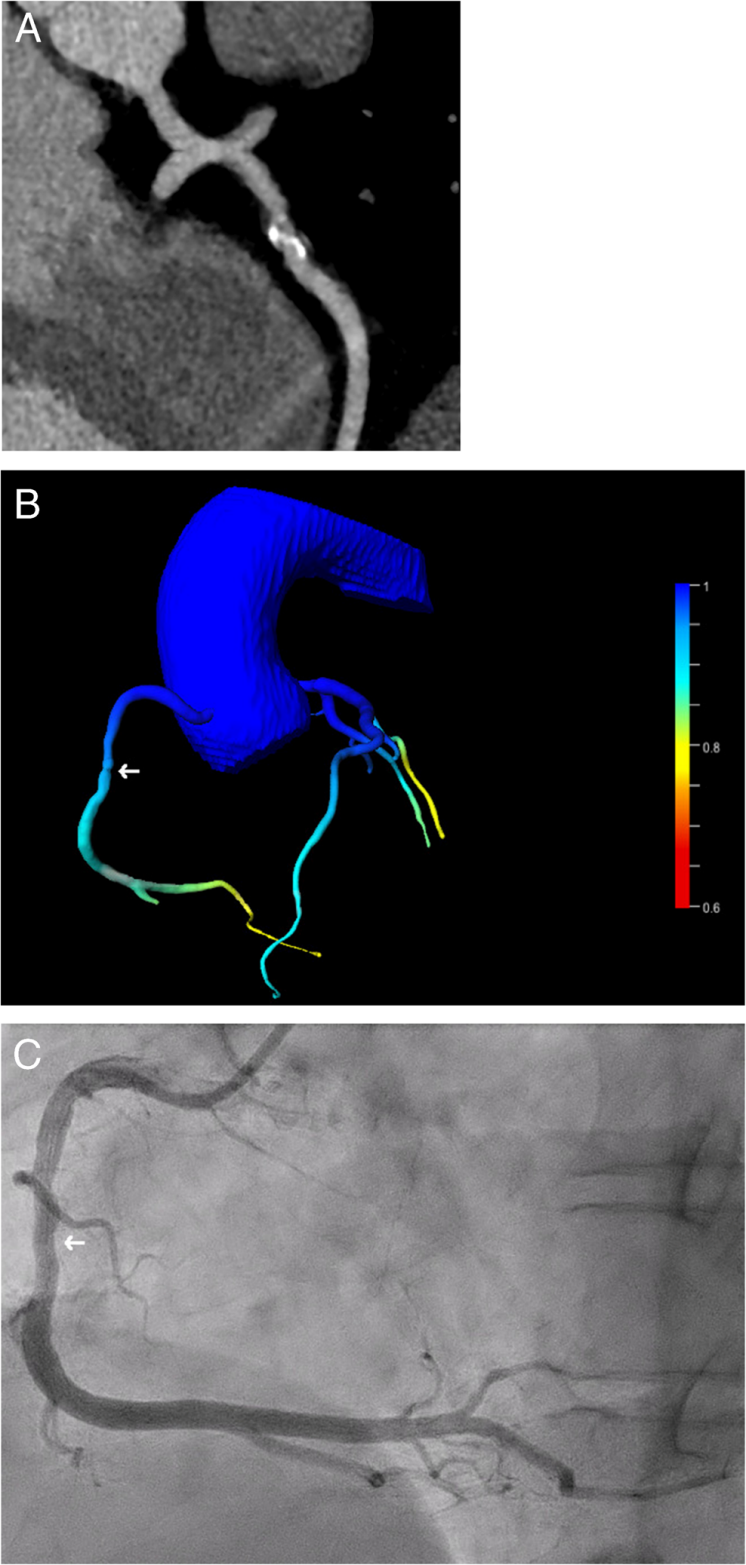
A 50-year-old male patient undergoing evaluation for liver transplantation. (**A**) Curved multiplanar reconstruction of the CCTA: A significant stenosis of the RCA was suspected (vessel lumen pre-stenosis 10.4 mm^2^, in-stenosis 4.5 mm^2^, post-stenosis 10 mm^2^); (**B**) Calculation of CT-FFR revealed hemodynamically non-significant stenosis (→, CT-FFR 0.87); (**C**) This finding was confirmed in ICA (→, iFR 0.97)

**Fig. 3:**
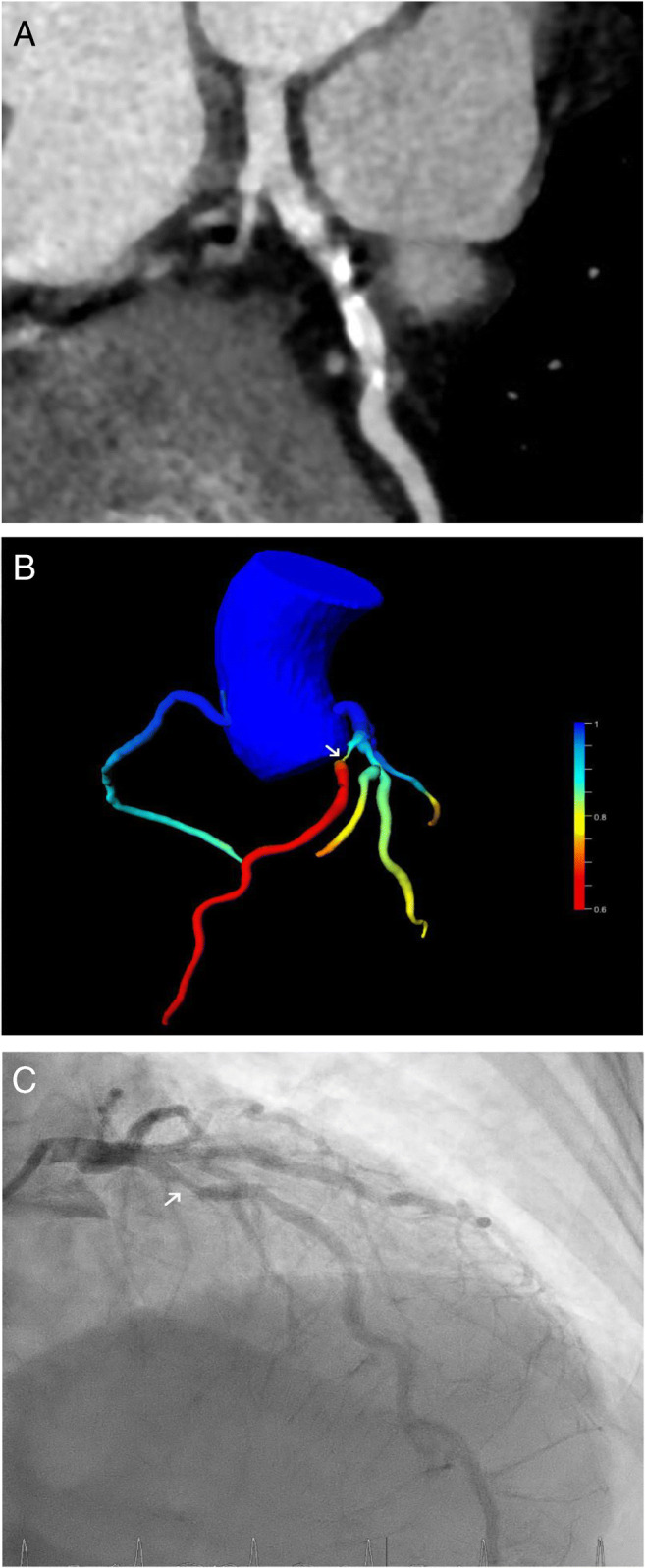
A 61-year-old female patient undergoing evaluation for liver transplantation. **A** Curved multiplanar reconstruction of the CCTA: A significant stenosis of the LAD was suspected (vessel lumen pre-stenosis 11.6 mm^2^, in-stenosis 4.2 mm^2^, post-stenosis 8.6 mm^2^); **B** Calculation of CT-FFR revealed hemodynamically significant stenosis (→, CT-FFR 0.43); **C** This finding was confirmed in ICA (→, iFR 0.7)

**Fig. 4:**
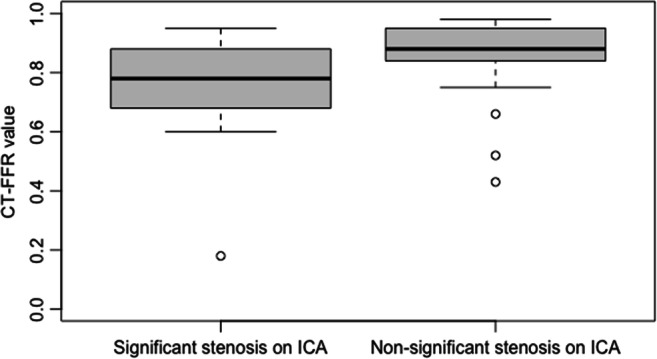
Boxplot of CT-FFR values in dependence of significant coronary stenosis in invasive catheter angiography. *CT-FFR* computed tomography derived fractional flow reserve*; ICA* invasive catheter angiography

In 57 lesions (76%), the CT-FFR was > 0.80 (Fig. 4); thus, the coronary stenosis was rated as “not hemodynamically significant.” In 52 of these lesions (91%), this finding was confirmed by ICA; in 36 of these lesions (69%), this was done by visual analysis alone, in 13 lesions (25%) the iFFR was determined, and in three lesions (6%) the iFR, respectively (Fig. 2). Therefore, in 69% of cases (52 of 75 lesions) with suspected coronary stenosis, additional analysis by CT-FFR correctly excluded the hemodynamic significance of the stenosis. Of the five lesions (9%) with a false-negative result, visual hemodynamically significant stenosis was present in three cases (60%), and in two additional lesions (40%), the hemodynamic significance of the stenosis was determined by iFFR. In 18 lesions (24%), the CT-FFR was ≤ 0.80; thus, the coronary stenosis was rated as “hemodynamically significant.” In 12 lesions (67%), this finding was confirmed by ICA; in eight of these lesions (67%), this was done by visual analysis alone, in two lesions (17%), hemodynamic significance was proven by FFR, and in two lesions (17%) by iFR (Fig. 3). Of the six lesions (33%) with a false-positive result, the hemodynamic significance of the stenosis was excluded by purely visual analysis (three cases) and by iFFR (three cases), respectively.

When CT-FFR ≤ 0.80 was used to determine the hemodynamic significance of a coronary artery stenosis (Fig. 4), sensitivity, specificity, PPV, and NPV were 71% (95%-CI 49–92%), 90% (95%-CI 82–98%), 67% (95%-CI 45–88%), and 91% (95%-CI 84–99%), respectively (Table 3). The diagnostic accuracy was 85% (95%-CI 85–86%).

**Table 3:** Contingency table for CT-FFR and ICA about detection of hemodynamically significant coronary stenosis

	Significant stenosis on ICA	No significant stenosis on ICA	
CT-FFR ≤ 0.80	*n* = 12	*n* = 6	PPV 0.67 (0.45–0.88)
CT-FFR > 0.80	*n* = 5	*n* = 52	NPV 0.91 (0.84–0.99)
	Sensitivity 0.71 (0.49–0.92)	Specificity 0.90 (0.82–0.98)	

*CT-FFR* computed tomography derived fractional flow reserve; *ICA* invasive coronary angiography

Confidence intervals are provided in brackets

## Discussion

At our liver transplant center, all patients are screened for cardiovascular risk factors during transplant evaluation. Our protocol for cardiac workup for liver transplantation candidates encompasses the following items: (1) BMI ≥ 35 kg/m^2^, (2) tobacco use, (3) diabetes (both insulin- and non-insulin-dependent), (4) status post coronary revascularization, and (5) status post-stroke. All patients older than 50 years or patients that meet at least 2 of the above-mentioned risk factors are undergoing further cardiac evaluation. For patients with known CAD, this is done using ICA as a standard of reference. Patients with low to intermediate likelihood of CAD or contraindication for ICA receive CCTA to rule out coronary artery disease. If the result is negative, further evaluation by ICA is not deemed necessary. Patients with suspected coronary stenosis receive invasive cardiac catheterization for further evaluation. While further evaluation of multivessel coronary artery disease by ICA before liver transplantation seems appropriate, the reduction of invasive evaluation in patients with intermediate but finally hemodynamically non-significant coronary stenoses would be desirable. In ICA, different techniques exist for the quantification of hemodynamically significant stenosis. For example, fractional flow reserve (iFFR) or instantaneous wave-free ratio (iFR) can be used for this purpose. iFFR is a pressure wire–based index and represented by the ratio of pressure distal of a coronary stenosis and the pressure in the proximal coronary artery using a hyperaemic agent to assure maximum achievable blood flow in the coronary arteries [15]. iFR is a method in which the measurement of intracoronary pressures before and after stenosis takes place in a special phase of diastole (the so-called wave-free period) and thus does not require the use of a hyperaemic agent [15]. For non-invasive imaging of the coronary arteries, commercial vendors have allowed the calculation of computed fractional flow reserve (CT-FFR) from CCTA datasets using computational fluid dynamic (CFD) modeling for several years. Studies have shown that the CFD-CT-FFR provides high diagnostic accuracy for the diagnosis of hemodynamically significant CAD compared with the invasively determined FFR (iFFR) as the standard of reference [16]. However, the disadvantage of this method based on CFD modeling is the high computational cost, which currently cannot be provided as an on-site solution for many institutions. A cost-effective and rapidly available on-site solution for the determination of CFD-CT-FFR from CCTA data sets is therefore currently not possible with this technique. Due to these limitations, several research groups have recently explored the calculation of CT-FFR from CCTA datasets using machine learning algorithms (CT-FFR). The machine learning–based algorithm used in this study has been compared with the gold standard of iFFR measurement in several other studies [17, 18], showing a significant improvement in the informative value of CCTA with respect to the detection of hemodynamically significant coronary stenosis.

In our study, the high specificity and high NPV show that CT-FFR is well suited to correctly identify intermediate / not hemodynamically significant coronary stenoses. In about 70% of our cases, stenoses, initially rated as high-grade by visual assessment of CCTA data sets, were rated as hemodynamically non-significant after targeted analysis by use of the CT-FFR algorithm, which was confirmed by ICA. Thus, in the future, the additional use of CT-FFR might result that in a relevant proportion of patients ICA could be dispensed with.

Other advantages of the on-site solution for CT-FFR calculation are the rapid availability and the short duration until the results are available. In our study, the time required for evaluation was not recorded separately, but other studies have evaluated the time required for evaluation with the algorithm we also used; Hu et al [18] required an average of 18 ± 7 (12–30) min for a single CT-FFR evaluation, a duration largely consistent with the authors’ personal experience. In our study, the speed of CT-FFR computation was particularly dependent on image quality. If the image quality was excellent (no motion, respiration, or pulsation artifacts and perfect contrasting of the coronary arteries), no relevant corrections of the automatically detected centerline or vessel contouring were necessary. However, if the image quality was not optimal (especially pulsation artifacts could not be completely avoided in some of our cases), manual corrections to the centerline or vessel contouring were necessary in some cases. A possible disadvantage for some users is the additional personal effort: the calculation of the CT-FFR requires additional post-processing of the CCTA data and in particular the checking and, if necessary, correction of the automatically generated centerline and the vessel contouring takes additional time. In contrast, the calculation of CFD-CT-FFR via commercial providers requires less personal effort for the user.

Our results are consistent with those of other research groups that have compared CT-FFR with the gold standard of invasively measured iFFR in different patient collectives: Coenen et al [17] showed in a large-scale multi-center study that CT-FFR helps to correctly classify hemodynamically nonsignificant coronary stenoses and that the CT-FFR performs equally well as the CFD-CT-FFR. Furthermore, Coenen et al [17] showed that the machine learning–based algorithm profits from optimal image quality. Hu et al [18] also demonstrated high diagnostic accuracy of CT-FFR for the detection of hemodynamically significant coronary stenoses and a statistically significant, strong correlation between CT-FFR and iFFR.

Our study has several limitations. First, patients being evaluated for LT are a very specific patient population with many comorbidities; furthermore, possible candidates may have been excluded during evaluation for LT for various reasons prior to receiving a CCTA for ruling out or confirming CAD; findings and results in this patient population cannot and should not be easily extrapolated to other collectives and an inclusion bias is possible. Second, patients with known CAD or high likelihood of CAD primarily underwent invasive evaluation by ICA at our center; they did not receive CCTA and are not included in this study. Third, not all patients who underwent ICA received measurements with iFFR or iFR; in fact, in most patients, hemodynamically significant stenosis was confirmed or excluded by visual analysis alone. Fourth, in this study, both iFFR and iFR were used if visual analysis using QCA was not sufficient to determine the hemodynamic significance of a coronary stenosis. In recent years, iFFR has been largely replaced by iFR at our institute but due to the recruitment period, both methods have been used in this study, though only in 2 patients that iFFR has been used. This could lead to a limited reproducibility of the results.

## Conclusion

Machine learning–based calculation of CT-FFR seems to be a very promising noninvasive approach for exclusion of hemodynamic significance of coronary stenoses in patients undergoing evaluation for liver transplantation and could help to reduce the rate of invasive coronary angiography in this high-risk population.

## Abbreviations

CAD: Coronary artery disease
CCTA: Coronary computed tomography angiography
CFD: Computational fluid dynamics
CT-FFR: Computed tomography derived fractional flow reserve
FFR: Fractional flow reserve
ICA: Invasive coronary angiography
iFFR: Invasive fractional flow reserve
iFR: Instantaneous wave-free ratio
LAD: Left anterior descending artery
LT: Liver transplantation
QCA: Quantitative coronary angiography
RCA: Right coronary artery
RCX: Ramus circumflex artery

## Declarations

### Guarantor

The scientific guarantor of this publication is Thomas W. Schlosser.

### Statistics and biometry

No complex statistical methods were necessary for this paper.

### Methodology

• retrospective

• observational

• performed at one institution
